# Indirect Estimation of Absorbed Infrared LED Radiant Power Using Contactless Thermal Sensing

**DOI:** 10.3390/s26134055

**Published:** 2026-06-26

**Authors:** Sorin Eugen Popa, Petru Gabriel Puiu, Dragoș Alexandru Andrioaia, Roxana Margareta Grigore, Ramona Lenuța Avădanei

**Affiliations:** The Department of Power Engineering and Computer Science, Faculty of Engineering, “Vasile Alecsandri” University of Bacau, 600115 Bacau, Romania; ppgabriel@ub.ro (P.G.P.); dragos.andrioaia@ub.ro (D.A.A.); rgrigore@ub.ro (R.M.G.); ramona-lenuta.avadanei22@ing.ub.ro (R.L.A.)

**Keywords:** NIR LED, radiant power, calorimetry, contactless infrared temperature measurement, low-cost instrumentation

## Abstract

The accurate characterization of low-power near-infrared LEDs typically requires costly radiometric equipment, limiting broader accessibility. This study proposes a low-cost indirect method for comparative NIR LED characterization based on the thermal response of black-coated aluminum absorbing targets monitored by a commercial MLX90614 contactless temperature sensor integrated with an ESP32 acquisition system. The absorbed optical power was estimated from a steady-state energy-balance model combining convective and radiative heat transfer, with geometry-dependent effective coefficients derived for 10 mm and 15 mm diameter targets. Experiments were conducted using 850 nm and 940 nm LEDs at drive currents between 30 mA and 100 mA. The absorbed power increased linearly with the drive current and electrical input power across all configurations, with *R*^2^ values of 0.995–0.997 and 0.996–0.999, respectively. The 15 mm targets exhibited higher capture ratios (10.4–11.9%) compared to the 10 mm targets (8.4–9.4%). The combined measurement uncertainty ranged from 13% at high drive currents to nearly 70% at low drive currents, with the temperature-rise sensitivity being the dominant factor; within the recommended operating range (≥70 mA for 10 mm and ≥80 mA for 15 mm targets), the uncertainty remained below 25%. The proposed platform enables reliable comparative characterization of low-power NIR emitters using exclusively off-the-shelf components.

## 1. Introduction

Infrared light-emitting diodes (IR LEDs) operating at 850 nm and 940 nm are widely employed in proximity sensing, optical communications, biomedical instrumentation, human–machine interfaces, and industrial automation [1,2,3]. In these applications, accurate knowledge of the emitted radiant power is essential for device calibration, performance optimization, energy management, and long-term reliability assessments. However, conventional radiometric characterization methods typically rely on calibrated photodiodes, thermopile detectors, integrating spheres, or electrical-substitution radiometers [4,5], all of which require expensive laboratory-grade instrumentation and controlled operating environments that limit their use in compact embedded or low-budget systems.

Thermal-based indirect estimation has emerged as a practical alternative. By monitoring the temperature rise of an absorbing target exposed to radiation, the optical power can be inferred from a steady-state energy-balance model without specialized radiometric hardware [6,7,8]. Such steady-state approaches inherently trade response speed for hardware simplicity, since reaching thermal equilibrium can require tens to hundreds of seconds, and their accuracy depends critically on the validity of the assumed convective and radiative heat-transfer coefficients. Strąkowska et al. demonstrated the feasibility of this approach using infrared thermography to correlate spatial temperature distributions with radiant-flux predictions [6,7], while Kim et al. implemented a compact photothermal sensor combining differential optical absorption with thermocouple measurements for high-power LED characterization [9]. Hegedüs et al. further showed that thermally coupled methods can reveal LED degradation and reliability behavior under varying operating conditions [10]. Despite these advances, the reported implementations rely either on infrared imaging cameras or custom-fabricated sensors, which limits their accessibility in low-cost embedded environments.

Advances in contactless infrared thermometry have produced compact, factory-calibrated sensors such as the MLX90614 and MLX90632, which offer a high thermal sensitivity, low power consumption, and straightforward digital integration [11,12]. Although these devices are extensively used in medical thermometry, industrial monitoring, and IoT systems, their potential for indirect optical-power estimation has not been systematically investigated. To the best of the authors’ knowledge, no prior studies have integrated a commercial MLX-series infrared thermometer into a calorimetric framework for NIR LED radiant-power characterization.

This work addresses that gap. The principal aim was to develop and experimentally validate a compact, low-cost platform for the indirect comparative characterization of low-power NIR LEDs, using a commercial MLX90614 sensor, thermally isolated black-coated aluminum absorbing targets, and an ESP32-based acquisition system. Experiments were conducted with 850 nm and 940 nm LEDs driven between 30 mA and 100 mA, using targets with a 10 mm or 15 mm diameter, to evaluate the sensitivity, repeatability, stabilization behavior, and correlation between the absorbed thermal power and the LED operating parameters. The method does not provide SI-traceable absolute radiometric measurements; rather, it offers a reproducible and embedded-compatible solution for a relative evaluation of low-power NIR emitters within a well-defined operating envelope.

## 2. Materials and Methods

### 2.1. Theoretical Framework and Physical Principle

The estimation of NIR LED radiant power is based on a calorimetric approach in which a black-coated aluminum absorbing target is heated by incident infrared radiation. Under steady-state conditions, the absorbed optical power ($P_{abs}$) is balanced by the heat losses to the surrounding environment through convection, radiation, and conduction, as expressed in Equation (1).

(1) $$P_{abs}=P_{conv}+P_{rad}+P_{cond}$$

Convective heat transfer (*P*_conv_) is estimated using Newton’s law of cooling, shown in Equation (2).

(2) $$P_{conv}=h\cdot A\cdot \left(T_{target}-T_{amb}\right)$$

where *h* represents the effective convective heat transfer coefficient, *A* is the exposed target area, and (*T*_target_–*T*_amb_) is the steady-state temperature difference between the absorbing target and the ambient environment.

Free convection in air is commonly characterized by average coefficients in the range of 5–10 W/m^2^K [13]. However, a preliminary analysis of the experimental results revealed measurable geometry-dependent differences between the two target sizes. Consequently, geometry-specific effective coefficients were adopted: *h*_10_ ≈ 10.5 W/m^2^ K for the 10 mm targets and *h*_15_ ≈ 7.4 W/m^2^ K for the 15 mm targets. These should be interpreted as effective lumped thermal parameters specific to the present experimental configuration—including the target geometry, natural-convection behavior, and mounting conditions—and must not be regarded as universal physical constants. Their derivation is detailed in Supplementary Section S1.

Radiative heat loss (*P*_rad_) was computed from the Stefan–Boltzmann law, according to Equation (3).

(3) $$P_{rad}=\varepsilon \cdot \sigma \cdot A\cdot \left(T_{target}^{4}-T_{amb}^{4}\right)$$

where *σ* = 5.67 × 10^−8^ W/(m^2^K^4^) is the Stefan–Boltzmann constant and *ε* = 0.95 is the emissivity of the matte-black acrylic coating.

The total heat transfer surface area (*A* = 2*A*_disc_ + *A*_side_) is given by Equation (4).

(4) $$A=2\cdot \left(\pi \cdot r^{2}\right)+(2\cdot \pi \cdot r\cdot \delta )$$

where *r* is the radius of the disk and *δ* = 0.1 mm is the height of the disk.

Conductive losses (*P*_cond_) through the three-point PLA support pins are minimized by design: the contact area is less than 0.5 mm^2^, and the low thermal conductivity of PLA (≈0.13 W/mK) [14,15] results in conduction losses below 1–2% of the total heat flux, which are therefore considered negligible. The primary objective of the model is to correlate the electrical input power (*P*_el_ *= V*_f_ · *I*_f_) with the experimentally estimated absorbed thermal power *P*_abs_ for comparative characterization of the investigated LEDs.

### 2.2. Hardware Design and Experimental Setup

#### 2.2.1. Measurement Platform

**Figure 1:**
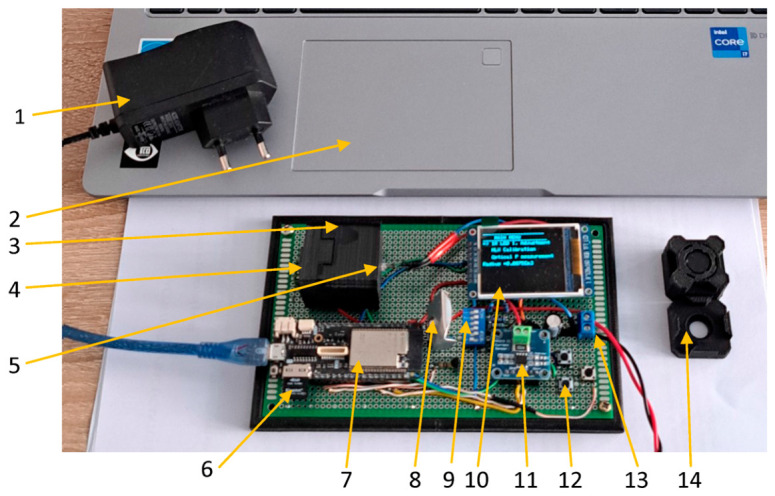
General assembly photo: (1) 9 V/1 A power supply; (2) laptop; (3) main experimental assembly holding the LED, target, and MLX sensor; (4) MLX90614 contactless IR sensor; (5) infrared LED under test; (6) SD-card; (7) ESP32—Lolin32Pro; (8) LED current source LM317; (9) DIP-switches for current selection; (10) TFT display; (11) INA219 current and voltage sensor; (12) push buttons for menu selection; (13) 9 V socket; and (14) main experimental assembly holding for 10 mm target.

**Figure 2:**
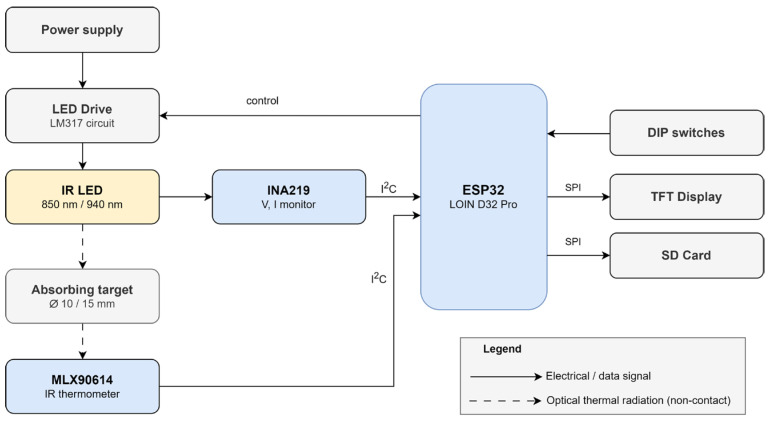
System-level block diagram of the measurement setup.

**Figure 3:**
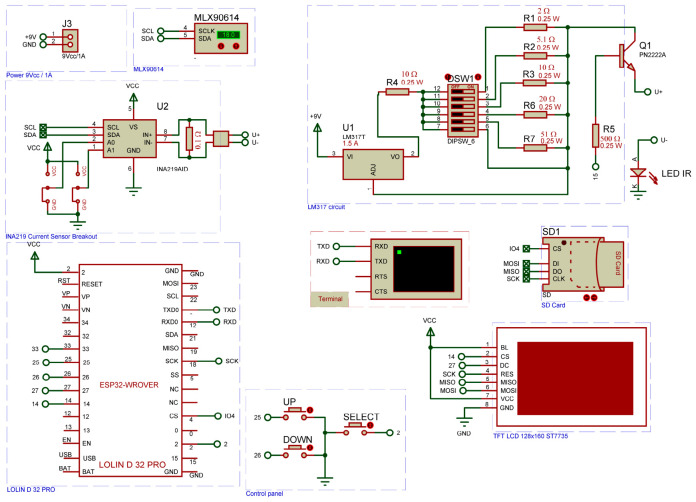
Schematic diagram of the experimental control and data acquisition system.

The experimental platform integrates an infrared excitation source, electrical monitoring circuitry, a non-contact infrared temperature sensor, and a microcontroller-based acquisition system. An overview of the assembly and system architecture is presented in Figure 1, Figure 2 and Figure 3.

A complete system-level block diagram of the measurement platform is shown in Figure 2.

The system is centered on an ESP32 microcontroller (LOLIN D32 Pro), which coordinates LED actuation, sensor acquisition, steady-state detection, and data logging. Two infrared LEDs were investigated: OSI3NA5111Y (850 nm) and OSI5CA5111Y (940 nm), both with a nominal viewing angle of 15° [16]. Table 1 presents the electro-optical characteristics of the used LED.

The LEDs were driven by an LM317-based constant-current source adjustable between 30 mA and 100 mA, controlled through transistor switching synchronized with the acquisition sequence. Electrical parameters were monitored in real time using an INA219 sensor over the I^2^C interface [17], enabling the acquisition of the LED current, voltage, and electrical input power. The target and ambient temperatures were measured using an MLX90614 infrared thermometer [11]. All data were sampled at 1 Hz and stored on a microSD card in the CSV format.

To reduce thermopile measurement noise while preserving the thermal transient, the temperature signal was processed using a first-order infinite impulse response (IIR) filter according to Equation (5).

(5) $$T_{avg}\left(n\right)=0.95\cdot T_{avg}\left(n-1\right)+0.05\cdot T_{target}\left(n\right)$$

The selected filter time constant (*τ*_filter_ = 1/(1 − 0.95) = 20 s at a 1 Hz sampling rate) is significantly smaller than the thermal time constant of the system (*τ*_thermal_ > 180 s), ensuring effective noise suppression without distorting the thermal transient or delaying steady-state detection. The selected filter coefficients provide effective smoothing without affecting steady-state convergence, consistent with commonly used approaches in thermopile-based sensing systems [9,18,19].

#### 2.2.2. Mechanical Alignment and Optical Geometry

**Figure 4:**
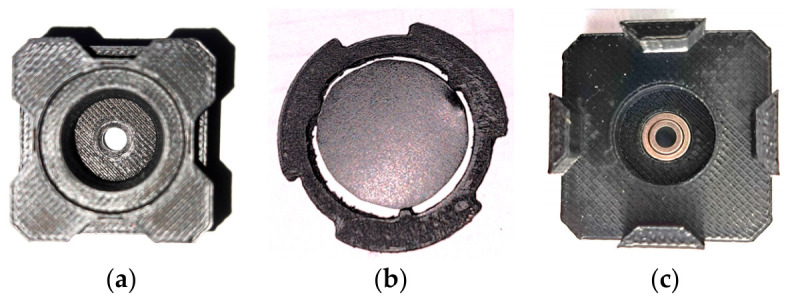
3D-printed PLA device for fixing the IR LED, the MLX sensor and the Al sample: (**a**) details of IR LED holder; (**b**) details of target holder; and (**c**) details of MLX sensor holder.

**Figure 5:**
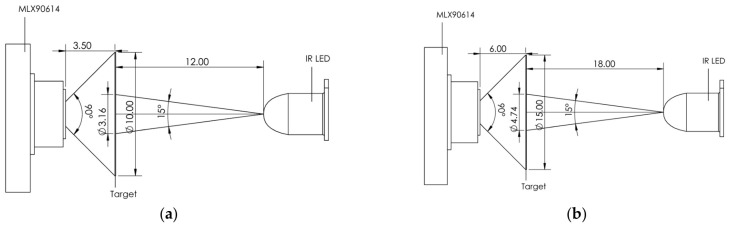
Optical alignment of the MLX sensor, target, and IR LED, including the distances between them for: (**a**) 10 mm target; (**b**) 15 mm target.

**Figure 6:**
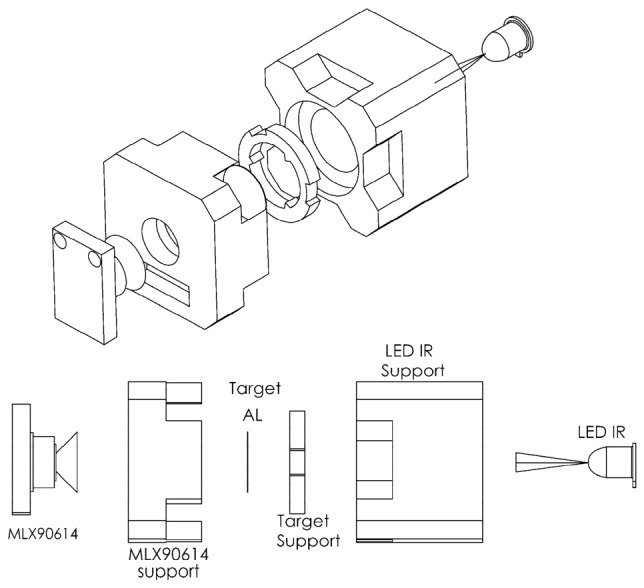
3D-printed modular support (PLA) designed with tolerances < 0.2 mm to maintain precise alignment between the NIR LED, the absorbing target, and the MLX90614 sensor.

A modular 3D-printed PLA fixture was designed to ensure stable and repeatable alignment between the LED, the absorbing target, and the MLX90614 sensor (Figure 4, Figure 5 and Figure 6). The mechanical tolerances were maintained below 0.2 mm to minimize geometric variations between experiments and to reduce stray reflections and external thermal disturbances.

The LED-to-target distances were selected based on the LED divergence angle and irradiance distribution: 12 mm for the 10 mm target and 18 mm for the 15 mm target, ensuring the complete and quasi-uniform illumination of the absorbing disk. The MLX90614 (optical class DAA, nominal FOV 90°) was positioned coaxially with the target at sensor-to-target distances of 3.5 mm and 6 mm for the 10 mm and 15 mm targets, respectively, ensuring that the measurement spot remained fully contained within the absorbing disk [11,20].

All measurements were performed under controlled indoor conditions at ambient temperatures between 23 °C and 25 °C. The assembly was enclosed to minimize air drafts and maintain a stable natural-convection regime.

#### 2.2.3. Absorbing Targets

Two circular aluminum disks—10 mm and 15 mm in diameter, and 0.1 mm thick—were used as absorbing targets. Aluminum was selected for its high thermal conductivity, which promotes rapid and uniform thermal equilibration across the target surface. Both disks were coated with matte-black acrylic paint, yielding an emissivity of *ε* ≈ 0.95, consistent with values reported for matte-black optical coatings on metallic substrates [21,22]. Each target was mounted on a dedicated PLA support using minimal point-contact fixation to reduce conductive losses to the mechanical structure.

The lateral surface area (*A*_side_ = 2·*π*·*r*·*δ*) contributes less than 0.4% of the total heat transfer area for both target sizes and is therefore negligible in the energy balance; it is retained in Equation (4) for completeness.

### 2.3. Software Implementation

#### 2.3.1. Firmware Architecture

**Figure 7:**
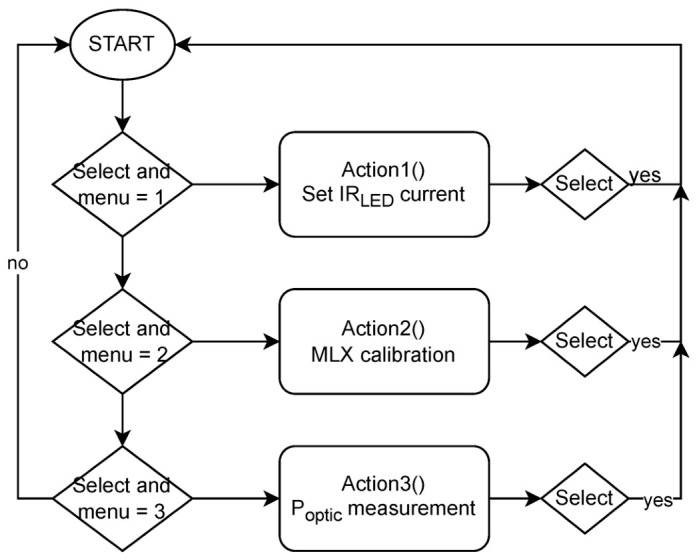
Logic flow of the temperature monitoring and steady-state detection algorithm.

The measurement platform was controlled by firmware implemented on the ESP32 using the Arduino IDE (version 2.3.7) development environment. The software performs synchronized LED control, sensor acquisition, thermal-state monitoring, and data logging, organized into three functional layers: user interaction, experimental control, and data acquisition and processing. The measurement algorithm is illustrated in Figure 7.

#### 2.3.2. Steady-State Detection

The absorbed optical power was evaluated only after the thermal system reached steady-state conditions. Equilibrium was identified when the derivative of the filtered temperature signal satisfied the criterion in Equation (6) for a predefined validation interval.

(6) |dT/dt|<0.005 °C/s

Two stability thresholds were used during the experimental sequence, as summarized in Table 2. During the initial thermal equilibration phase (LED OFF), a criterion of ∣*dT*/*dt*∣ < 0.015 °C/s sustained for 15 s was applied to confirm thermal equilibrium with the environment. During absorbed-power measurements (LED ON), a stricter threshold of ∣*dT*/*dt*∣ < 0.005 °C/s maintained for 60 s was used to ensure complete convergence of the calorimetric balance.

This approach minimized the influence of transient thermal fluctuations and improved the repeatability of the absorbed-power estimation [6,7].

#### 2.3.3. Data Acquisition and Logging

The temperature, current, and voltage were acquired at 1 Hz and stored as CSV files on the microSD card. Each record included timestamps, the electrical operating conditions, the ambient and target temperatures, and the calculated absorbed optical power. Five independent measurements were recorded for each experimental configuration—defined by the LED wavelength, target diameter, and drive current—to enable a repeatability evaluation.

#### 2.3.4. Operating Procedure

Each measurement followed three sequential stages:

- Current setting: the LED drive current was configured to the target value while electrical parameters were monitored in real time;
- Thermal equilibration: the system confirmed that the absorbing target had reached thermal equilibrium with the environment before activating the LED;
- Absorbed-power measurement: the LED was activated and the target temperature was monitored until steady-state conditions were detected, after which the stabilized data were recorded.

### 2.4. Uncertainty Analysis

The overall uncertainty of the indirect optical-power estimation originated from four sources: the temperature measurement, electrical measurement, geometric alignment, and thermal-model assumptions.

The dominant contributor was the temperature measurement uncertainty of the MLX90614, which provided a digital-interface measurement resolution of 0.02 °C and a typical accuracy of ±0.5 °C within the operating range [11,12]. Since *P*_abs_ was derived from the temperature difference Δ*T* = *T*_target_ − *T*_amb_, the propagated uncertainty in Δ*T* is approximately ±0.7 °C, primarily affecting the convective heat-transfer term.

The electrical uncertainty originated from the INA219 current/power monitor (typical maximum current-measurement error of ±1% over the operating temperature range) [17] combined with the ±1% tolerance of the 0.1 Ω shunt resistor used in the acquisition module; the resulting root-sum-square electrical uncertainty remained below ±2%, contributing less than 0.04 percentage points to the combined total uncertainty.

The geometric uncertainty was minimized by the rigid 3D-printed fixture (Section 2.2.2). Residual alignment errors, including nonuniform illumination and slight optical-axis deviations, were estimated to contribute approximately 3% to the absorbed-power uncertainty [4]. Surface emissivity variations contributed less than 2% for the matte-black coated targets used [21,22].

The model-related uncertainty arose from the geometry-dependent effective heat-transfer coefficients h_10_ and h_15_, which were derived from a simplified low-Rayleigh-number analysis. Deviations of the operating-point convective coefficient from the adopted fixed values reached up to 5.7% for the 15 mm target across the investigated current range (see Supplementary Section S1), propagating directly into the convective term of the energy balance.

The total combined uncertainty was estimated using the root-sum-square (RSS) method according to Equation (7).

(7) $$u_{total}=\sqrt{u_{T}^{2}+u_{el}^{2}+u_{geo}^{2}+u_{model}^{2}}$$

The resulting uncertainty was strongly dependent on the operating point. It ranged from approximately 13% at high drive currents on the 10 mm targets—where the temperature rise was largest—to nearly 70% at low drive currents on the 15 mm targets, where the small thermal rise (Δ*T* ≈ 1 °C) severely reduced the signal-to-noise ratio of the calorimetric measurement. Within the recommended operating range—drive currents of 70 mA and above for 10 mm targets, and 80 mA and above for 15 mm targets—the combined uncertainty remained below 20% and 25%, respectively. These values are consistent with previously reported calorimetric and photothermal characterization methods for low-power infrared LEDs [6,7,9], and reflect the inherent limitations of a point-temperature indirect approach rather than a deficiency of the measurement platform.

Given the dominant role of the ±0.5 °C MLX90614 temperature accuracy in the combined uncertainty budget, all uncertainties reported throughout this manuscript throughout this manuscriptare rounded to a single significant digit, with the corresponding mean values rounded to the same decimal place, following standard metrological reporting practice [23].

For the linear-fit parameters reported in the linear regression tables reported in Section 3 (slope and intercept of *P*_abs_ vs. *I*_led_, *P*_abs_ vs. *P*_el_, and capture ratio *η*_cap_), one additional significant figure is retained relative to the single-measurement precision of *P*_abs_. This is justified by the least-squares averaging over six to seven independent data points per configuration, which reduces the random component of the uncertainty by approximately $1/\sqrt{n}$ relative to a single measurement, and is consistent with standard regression-uncertainty propagation practice.

## 3. Results

### 3.1. Overview of the Experimental Dataset

A total of 140 experimental measurements were performed across four LED–target configurations: 850 nm/10 mm, 850 nm/15 mm, 940 nm/10 mm, and 940 nm/15 mm. For each configuration, seven drive-current levels were investigated, ranging from 30 mA to 100 mA. The measured quantities included the LED current and voltage, ambient temperature, target temperature rise, absorbed thermal power, convective and radiative power components, system capture ratio, and thermal stabilization time.

Steady-state conditions were automatically identified using the derivative-based criterion described in Section 2.3.2, corresponding to |*dT*/*dt*| < 0.005 °C/s sustained for 60 s. For each experiment, the reported steady-state quantities represent the mean of the final 60 stabilized samples across five independent repetitions.

The complete dataset, including all intermediate quantities (*I*_led_, *U*_led_, *T*_amb_, Δ*T*, *P*_conv_, *P*_rad_), is provided in Table S1 (Supplementary Materials); Table 3 summarizes the absorbed power *P*_abs_, which is the primary quantity of interest. Across all the investigated configurations, the absorbed power increased monotonically with the LED drive current. The relative standard deviation of *P*_abs_ remained below 5% for all configurations except the 850 nm/10 mm case at 30 mA (≈6.5%), where the small temperature rise (Δ*T* ≈ 1.6 °C) reduced the signal-to-noise ratio and increased measurement scatter.

A clear influence of target geometry was observed. For both wavelengths, the 15 mm targets consistently produced higher absorbed power values than the corresponding 10 mm configurations, demonstrating improved optical interception of the divergent LED beam. The radiative component remained approximately constant across all drive currents, accounting for approximately 35% of the total heat dissipated for the 10 mm targets and approximately 44% for the 15 mm targets. This geometry-dependent difference is a direct consequence of the distinct effective heat-transfer coefficients adopted for each absorber size (*h*_10_ = 10.5 W/m^2^K; *h*_15_ = 7.4 W/m^2^K): the lower convective coefficient of the larger target reduces the convective share and correspondingly increases the radiative fraction. For both target geometries, convective heat transfer remained the dominant dissipation mechanism within the investigated operating range.

### 3.2. Absorbed Power as a Function of Drive Current and Electrical Input Power

**Figure 8:**
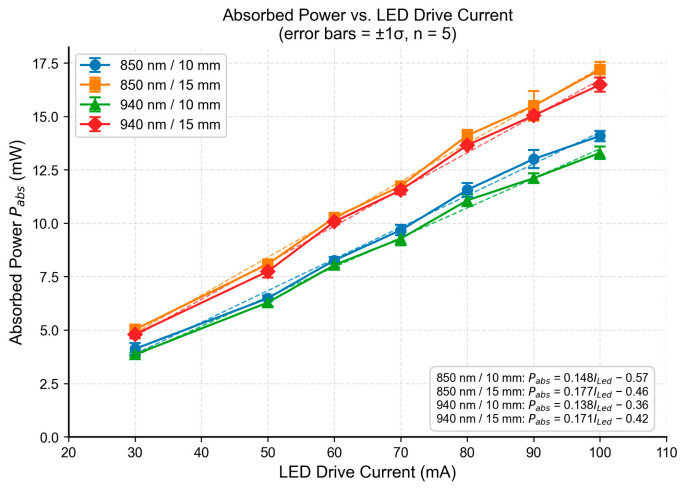
Absorbed power (*P*_abs_) as a function of the LED drive current for the four LED–target configurations. Dashed lines represent the linear regression fits.

The relationship between the absorbed thermal power (*P*_abs_) and the LED drive current is shown in Figure 8 for all investigated configurations, with the corresponding linear regression parameters summarized in Table 4.

A strong linear dependence was observed in all cases, with coefficients of determination ranging from *R*^2^ = 0.9953 to *R*^2^ = 0.9970. This behavior confirms that, within the investigated current range, the proposed calorimetric system operates in a stable linear regime without evidence of thermal saturation or significant nonlinear heat-transfer effects.

The regression slope provides a direct measure of the thermal capture response per unit of drive current. The 15 mm targets consistently exhibited larger slopes than the 10 mm configurations: the slope for 850 nm/15 mm exceeded that of 850 nm/10 mm by approximately 19%, reflecting the enhanced radiant interception afforded by the larger absorbing area. The non-zero intercepts were attributed primarily to the radiometric baseline of the MLX90614 sensor and residual thermal losses under low-power operating conditions; as these affected the intercept rather than the slope, they did not compromise the comparative analysis or the observed linearity.

**Figure 9:**
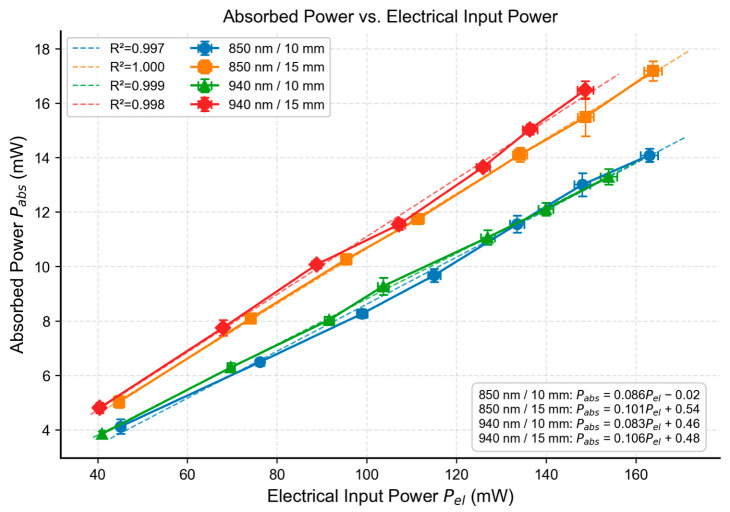
Absorbed power (*P*_abs_) as a function of the LED electrical power for the four LED–target configurations. Dashed lines represent the linear regression fits.

Figure 9 presents the relationship between the absorbed power and the electrical input power (*P*_el_ = *U*_LED_ · *I*_LED_). The corresponding regression parameters, summarized in Table 5, yielded even higher coefficients of determination (*R*^2^ > 0.996), confirming the strong electro-thermal correlation of the measurement platform.

The high linearity observed in both the *P*_abs_–*I*_LED_ and *P*_abs_–*P*_el_ relationships demonstrates that the proposed calorimetric approach provides a stable and reproducible comparative characterization of low-power NIR emitters across the full investigated operating range.

### 3.3. System Capture Ratio

**Figure 10:**
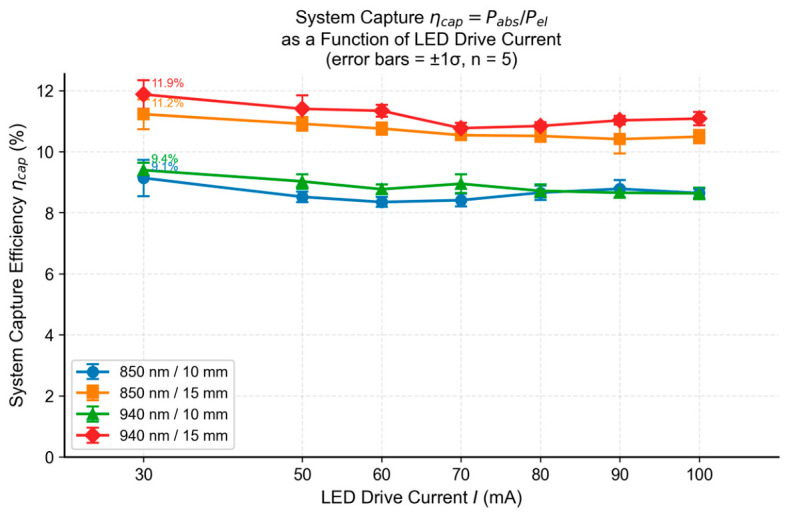
System capture ratio (*η*_cap_) as a function of the LED drive current for the four LED–target configurations.

The system capture ratio (*η*_cap_), defined as the ratio of the absorbed thermal power to the electrical input power (Equation (8)), is presented as a function of the drive current in Figure 10 and Table 6.

(8) $$\eta_{cap}=\frac{P_{abs}}{P_{el}}$$

The results reveal a clear separation between the 10 mm and 15 mm target configurations across all operating points. The 15 mm targets consistently achieved higher capture ratios due to improved interception of the divergent optical beam. Among all configurations, the 940 nm/15 mm arrangement exhibited the highest values, reaching 11.87% at 30 mA and stabilizing between approximately 10.8% and 11.1% at higher currents. The 10 mm targets remained below 9.5% across the full current range. The slight decrease in *η*_cap_ with increasing drive current observed for most configurations was attributed to the nonlinear increase in *P*_el_ relative to *P*_abs_ at higher junction temperatures. Despite these variations, *η*_cap_ remained relatively stable within each configuration, confirming a consistent calorimetric behavior once steady-state conditions were achieved.

### 3.4. Thermal Stabilization Time

**Figure 11:**
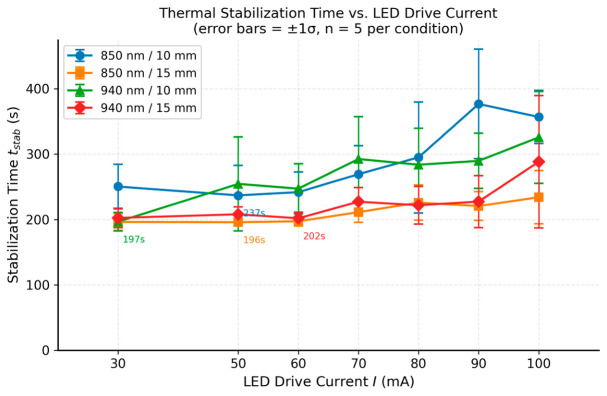
Thermal stabilization time (*t*_stab_) as a function of LED drive current for the four LED–target configurations.

The stabilization time required to satisfy the steady-state criterion is presented in Figure 11 as a function of the LED drive current for all the investigated configurations.

Across the complete dataset, the stabilization times ranged from approximately 186 s to 430 s depending on the target geometry and operating conditions. Two distinct behaviors were observed.

The 15 mm targets exhibited relatively stable and moderate stabilization times across the full current range—between approximately 190 s and 290 s for 850 nm/15 mm, and up to 288 s for 940 nm/15 mm at 100 mA. The larger thermal mass of the 15 mm disks acted as a low-pass thermal filter, damping short-term convective fluctuations and promoting smoother convergence toward a steady state.

In contrast, the 10 mm targets exhibited a progressive increase in the stabilization time at higher drive currents, reaching up to 430 s for the 850 nm/10 mm configuration at 100 mA. At elevated absorbed-power densities, the smaller targets showed an increased sensitivity to local convective disturbances, generating temperature fluctuations that delayed satisfaction of the derivative-based stabilization criterion. The associated increase in the standard deviation at high currents further supports this interpretation, particularly for the 850 nm/10 mm configuration above 80 mA.

These results indicate that the 15 mm targets not only improve thermal capture, but also provide more stable and faster steady-state convergence under natural-convection conditions.

### 3.5. Thermal Power Decomposition

**Figure 12:**
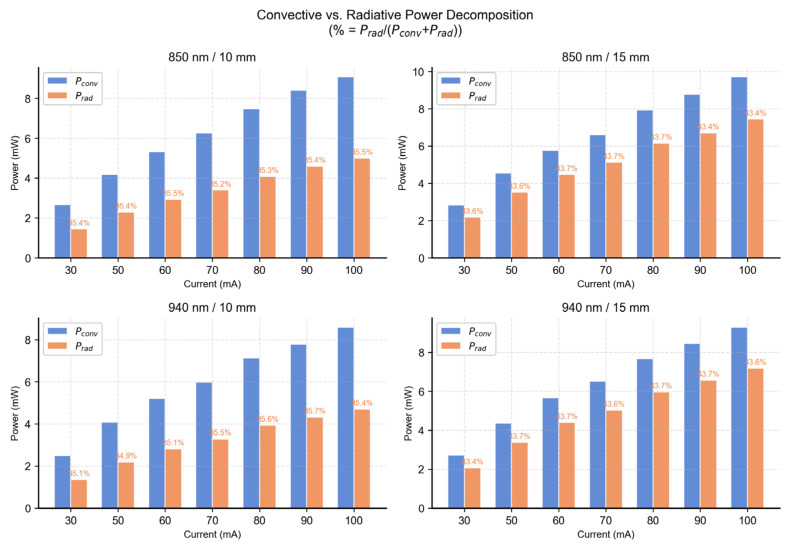
Decomposition of absorbed thermal power into convective and radiative components for all LED–target configurations.

Figure 12 presents the decomposition of the absorbed thermal power into convective and radiative components for all configurations and drive currents.

For all the investigated operating conditions, convective heat transfer represented the dominant contribution. The radiative fraction remained approximately constant across the full current range, at approximately 35% for the 10 mm targets and approximately 44% for the 15 mm targets. This geometry-dependent difference, already noted in Section 3.1, is a direct consequence of the distinct effective heat-transfer coefficients and exposed surface areas associated with each target size. The stability of the radiative fraction with the drive current confirms that the thermal operating regime remained within the linear free-convection domain throughout the experiments, with no evidence of nonlinear heat-transfer transitions.

**Figure 13:**
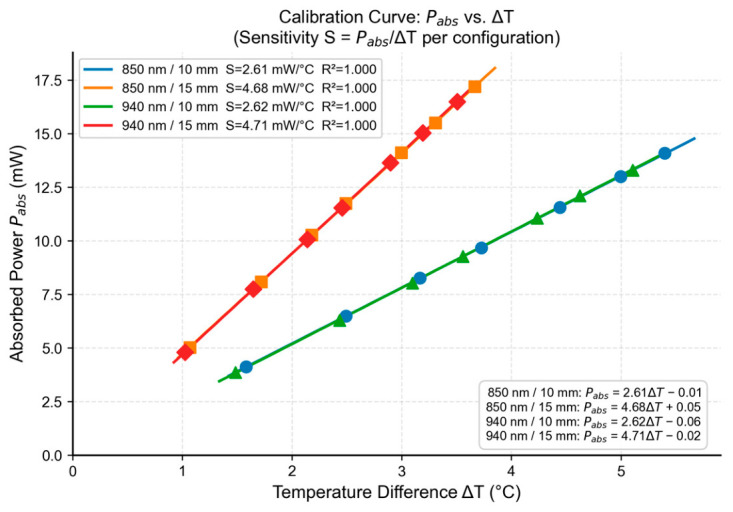
Absorbed thermal power (*P*_abs_) as a function of steady-state target temperature rise (Δ*T*) for all four LED–target configurations.

Figure 13 further illustrates the relationship between the absorbed power and the steady-state temperature rise Δ*T* for all four configurations. A strong linear dependence was observed in all cases, with *R*^2^ = 1.000, confirming that Δ*T* serves as a reliable calorimetric indicator of the absorbed optical power. The 15 mm targets exhibited an approximately 80% higher thermal sensitivity (*S* ≈ 4.7 mW/°C) compared to the 10 mm targets (*S* ≈ 2.6 mW/°C), directly reflecting their larger absorbing area and improved radiant interception. Under the small temperature excursions investigated (Δ*T* < 6 °C), both the convective term (*P*_conv_ = *h* · *A* · Δ*T*) and the radiative term can be locally linearized around the ambient temperature, which explains the near-perfect linearity of the *P*_abs_–Δ*T* calibration curves. The observed thermal balance validates the applicability of the steady-state radiative–convective model used for absorbed-power estimation.

### 3.6. Representative Transient Dynamics

**Figure 14:**
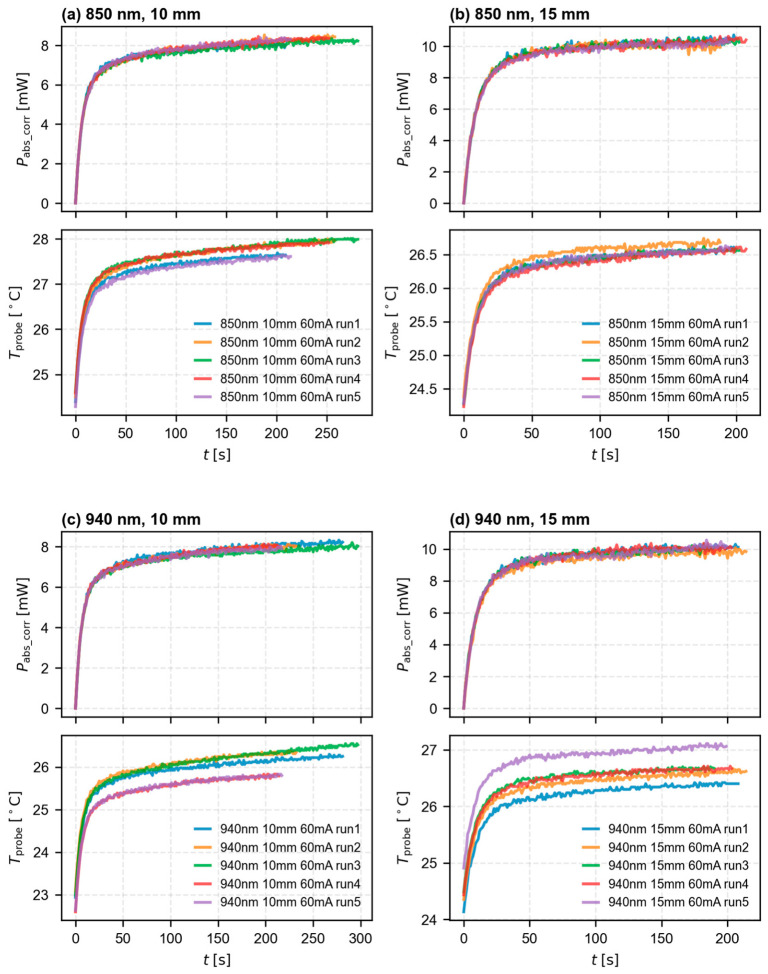
Transient thermal responses at 60 mA drive current for all four LED–target configurations: (**a**) 850 nm/10 mm; (**b**) 850 nm/15 mm; (**c**) 940 nm/10 mm; and (**d**) 940 nm/15 mm.

Representative transient responses recorded at 60 mA are presented in Figure 14 for all four configurations.

All the measured temperature profiles exhibited the characteristic exponential thermal response: an initial rapid heating phase followed by gradual convergence toward a steady state, with no evidence of thermal oscillations or instability. The 15 mm configurations showed slower, but smoother, thermal responses, consistent with their higher thermal capacitance and inertia. The 10 mm targets responded more rapidly, but displayed increased short-term fluctuations near the steady state, indicating a higher sensitivity to ambient convective disturbances.

The operating point of 60 mA provides a favorable compromise between a measurable temperature rise, an acceptable stabilization time, and reduced thermal scatter. At lower currents, the small ΔT amplifies the sensitivity to environmental perturbations, while at higher currents, the increased absorbed-power density exacerbates convection-related variability, particularly for the 10 mm targets.

### 3.7. Repeatability and Environmental Sensitivity

**Figure 15:**
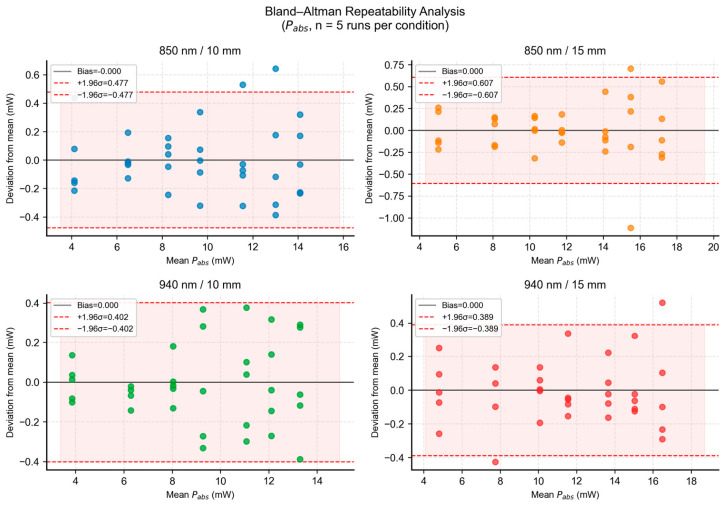
Bland–Altman repeatability analysis for the proposed calorimetric platform.

Figure 15 presents the Bland–Altman analysis of measurement repeatability across all configurations. The large majority of experimental points fell within the 95% limits of agreement, indicating consistent behavior between repeated measurements and stable operation of the acquisition system. Slightly larger deviations at higher absorbed-power levels were attributed to environmental convective fluctuations, which became more pronounced at elevated target temperatures.

**Figure 16:**
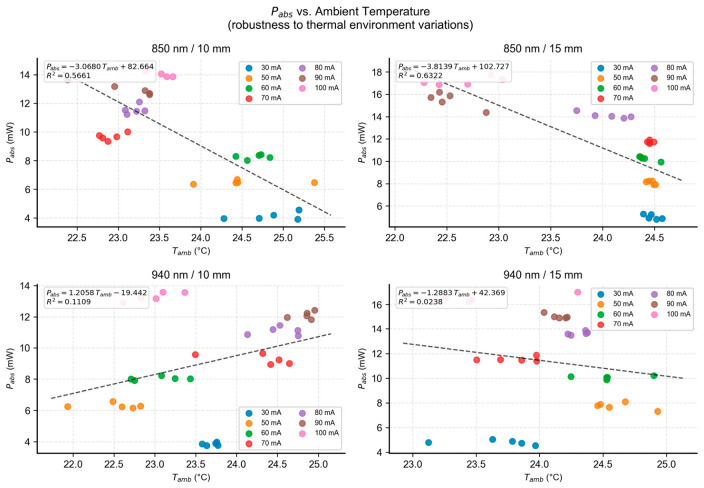
Variation in estimated absorbed power with ambient temperature for all configurations.

Figure 16 illustrates the sensitivity of the absorbed-power estimation to ambient temperature variations. No systematic drift was observed within the investigated indoor temperature range (approximately 22–25 °C), confirming an adequate stability under normal laboratory conditions. The 10 mm targets exhibited a moderately larger dispersion, consistent with their lower thermal inertia and higher sensitivity to local convective variability. The 15 mm targets showed a more stable response across the ambient temperature range, with reduced scatter attributable to their larger thermal mass. These results confirm that ambient convection represents the dominant environmental uncertainty source, particularly at low absorbed-power levels where small thermal perturbations become proportionally more significant.

### 3.8. Comparison with Manufacturer Datasheet

**Figure 17:**
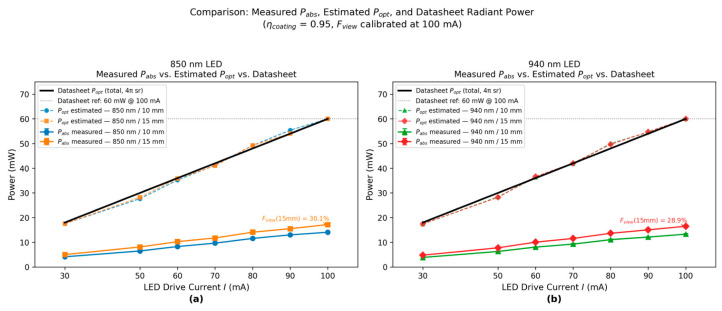
Comparison of experimentally measured absorbed power with manufacturer datasheet radiant power: (**a**) 850 nm LED; (**b**) 940 nm LED.

Figure 17 and Table 7 compare the experimentally measured absorbed thermal power with the nominal radiant characteristics provided in the LED manufacturer datasheet [16].

The nominal radiant power of both LEDs was 60 mW at 100 mA. The experimentally measured absorbed power values—ranging from 13.3 mW to 17.2 mW depending on the wavelength and target geometry—were substantially lower, as expected. The proposed platform measured only the fraction of emitted radiation geometrically intercepted and thermally absorbed by the target surface; the difference with the nominal figure reflects limited beam interception, finite target dimensions, angular emission characteristics, partial surface reflectance, and steady-state thermal losses rather than any deficiency of the measurement method. The 15 mm targets consistently yielded a higher absorbed power and larger capture ratios than the 10 mm configurations, confirming the dominant influence of the target geometry on radiant interception. The reported *η*_cap_ values must not be interpreted as the LED electro-optical conversion efficiency.

## 4. Discussion

The experimental results demonstrate that the proposed calorimetric platform provided stable and reproducible comparative characterization of low-power NIR LEDs across the full investigated operating range. Several consistent trends were identified across all four LED–target configurations.

The absorbed thermal power exhibited a strong linear dependence on both the LED drive current and the electrical input power, with coefficients of determination of *R*^2^ = 0.995–0.997 for the *P*_abs_–*I*_LED_ relationship and *R*^2^ = 0.996–0.999 for the *P*_abs_–*P*_el_ relationship. This behavior confirms that the system operates within a quasi-linear thermal regime without evidence of saturation or abrupt nonlinear heat-transfer effects, consistent with the expected behavior of low-power semiconductor emitters operating below thermal roll-off conditions. The non-zero intercepts of the *P*_abs_–*I*_LED_ regressions were attributed to the radiometric baseline of the MLX90614 sensor and residual thermal losses at low operating levels; since these affect the intercept rather than the slope, they do not compromise the comparative validity of the method.

The target geometry emerged as the dominant design parameter. The 15 mm absorbing targets systematically produced higher absorbed-power values and larger system capture ratios (10.4–11.9%) compared to the 10 mm configurations (8.4–9.4%), reflecting the improved solid-angle interception of the divergent LED beam by the larger absorbing surface. The slight decrease in *η*_cap_ with increasing drive current, observed in most configurations, is consistent with the superlinear increase in the electrical input power relative to the absorbed thermal power at higher junction temperatures, a behavior commonly reported in characterization studies of low-power infrared emitters [9,24]. Although the proposed methodology does not directly measure the junction temperature or emitted radiant flux, the calorimetric response may indirectly reflect temperature-dependent variations in the LED electro-optical behavior through changes in the absorbed thermal power.

The thermal decomposition analysis confirmed that convective heat transfer remained the dominant dissipation mechanism for all configurations throughout the entire investigated current range. The radiative fraction was approximately stable at ~35% for the 10 mm targets and ~44% for the 15 mm targets—a geometry-dependent difference that is a direct consequence of the distinct effective heat-transfer coefficients and exposed surface areas associated with each target size. The stability of this thermal partition across all operating conditions confirms that the system remained within a stable natural-convection regime and validates the applicability of the steady-state radiative–convective model adopted for absorbed-power estimation.

Regarding transient behavior, the 15 mm targets consistently exhibited smoother and more stable convergence toward a steady state, with stabilization times ranging between approximately 190 s and 290 s. The 10 mm targets showed a progressive increase in the stabilization time at higher drive currents, reaching up to 430 s for the 850 nm configuration at 100 mA, attributed to an increased sensitivity to local convective disturbances at elevated absorbed-power densities. These results indicate that the 15 mm targets not only improved thermal capture, but also provided more consistent stabilization dynamics under natural-convection conditions.

Moderate differences in the absorbed power between the 850 nm and 940 nm configurations were observed under otherwise identical operating conditions. These variations were attributed to differences in LED emission characteristics, beam divergence, and the spectral absorptivity of the matte-black coating, which was not perfectly uniform across the near-infrared spectrum. Wavelength-dependent variations in the absorbed thermal power at this level were therefore expected and did not indicate a systematic measurement artifact.

### 4.1. Comparison with Related Thermal-Based Methods

Table 8 summarizes the key characteristics of the proposed method in relation to the two most relevant prior works on thermal-based LED optical power estimation. Strąkowska et al. [6,7] demonstrated that IR thermographic imaging can reliably estimate LED optical power by simultaneous temperature measurements of an LED and a reference resistor under identical thermal boundary conditions, validated against an integrating sphere. While their approach achieves a high confidence through absolute calibration, it requires laboratory-grade IR camera equipment, which limits its applicability in embedded or low-cost environments. Kim et al. [9] proposed a compact photothermal sensor based on a pair of sheet-type thermocouples with different optical absorptivities, achieving a maximum error of 3% against an integrating sphere reference at 645 mW—the highest accuracy among the compared methods. However, their sensor requires custom fabrication and is optimized for high-power LEDs, making it less suitable for the low-power NIR range addressed in this work (1.4–17.2 mW absorbed).

The proposed method occupies a distinct and complementary niche. Implementations based on integrating spheres [25,26,27] or calibrated photodiode systems provide SI-traceable absolute radiant-flux measurements, but require dedicated radiometric infrastructure. Thermal imaging approaches such as those reported by Strąkowska et al. [6,7] offer spatially resolved flux estimation, but depend on infrared cameras and complex inverse models. The present platform uses exclusively commercial off-the-shelf components (MLX90614, INA219, ESP32), operates without external reference instrumentation, and provides fully autonomous data acquisition via an on-board microSD module, sacrificing absolute traceability in favor of hardware simplicity, embedded compatibility, and a low cost. The combined measurement uncertainty ranges from approximately 13% at high drive currents on the 10 mm targets to nearly 70% at low drive currents on the 15 mm targets—higher than that of Kim et al. [9], but within the typical range reported for calorimetric and photothermal LED characterization methods [6,7,8,9], and reduced to below 20% for 10 mm targets and below 25% for 15 mm targets within the recommended operating envelope (≥70 mA and ≥80 mA, respectively). The method offers a superior accessibility, portability, and spectral robustness across the 850–940 nm NIR band, where neither of the compared methods has been specifically evaluated. To the best of the authors’ knowledge, no prior work has integrated a commercial MLX-series contactless thermometer into a calorimetric framework for NIR LED characterization, representing the principal novelty of this contribution.

### 4.2. Limitations and Future Work

Several limitations of the present implementation should be acknowledged. The effective heat-transfer coefficients adopted in the thermal model (*h*_10_ ≈ 10.5 W/m^2^K, *h*_15_ ≈ 7.4 W/m^2^K) are configuration-specific lumped parameters that incorporate the natural convection, target geometry, mounting conditions, and local environmental variability; they must not be interpreted as universal physical constants and may require re-derivation if the mechanical layout or operating environment changes substantially. Outside the recommended operating envelope, the results retain qualitative and comparative value, but should not be used for quantitative absorbed-power estimations, given the dominant contribution of the temperature-measurement sensitivity at low thermal rises. The stabilization times observed—ranging from approximately 186 s to 430 s depending on the target geometry and operating point—may constrain the throughput in applications requiring rapid sequential characterization. Finally, the absence of external radiometric calibration prevents direct traceable conversion between the absorbed thermal power and the emitted radiant flux.

It should also be noted that the present characterization is restricted to steady-state behavior; a full assessment of the system’s dynamic response—including thermal time constants and transient settling behavior—falls outside the present scope, but is being addressed in ongoing work using lumped-parameter RC thermal models validated against axisymmetric finite-element simulations of the absorbing targets.

Future work will focus on three main directions: the validation of absorbed-power estimates against a calibrated reference optical power meter, such as an integrating-sphere radiometric setup, to establish the absolute accuracy limits of the method; the optimization of the target geometry and thermal mass to reduce the stabilization time while maintaining an adequate signal-to-noise ratio at low drive currents; and the implementation of active thermal compensation or forced-convection stabilization to reduce environmental sensitivity and extend the valid operating envelope toward lower drive currents.

## 5. Conclusions

This study presented and experimentally validated a compact, low-cost calorimetric platform for the comparative characterization of low-power near-infrared LEDs. The system integrates a commercial MLX90614 contactless infrared thermometer, thermally isolated black-coated aluminum absorbing targets of 10 mm and 15 mm diameters, and an ESP32-based embedded acquisition system with derivative-based steady-state detection. To the best of the authors’ knowledge, this represents the first reported integration of a commercial MLX-series infrared thermometer into a calorimetric framework for NIR LED radiant-power characterization.

Across all four LED–target configurations, the platform demonstrated stable, linear, and reproducible calorimetric behavior, with the target geometry emerging as the dominant design parameter governing both absorbed-power capture and thermal stabilization dynamics. Although the proposed methodology does not provide SI-traceable radiometric measurements and its combined uncertainty remains strongly dependent on the operating point, the results confirm that it constitutes a practical and reproducible solution for the relative evaluation of low-power NIR emitters using exclusively off-the-shelf components. The reduced hardware complexity and compact embedded architecture make the platform suitable for applied comparative characterization, experimental diagnostics, and educational measurement systems.

Future work will focus on validating the absorbed-power estimates against a calibrated reference instrument and on extending the system’s operating envelope and dynamic response through improved target design and active thermal compensation, broadening its applicability to a wider range of NIR sources and operating conditions.

## Figures and Tables

**Table 1:** Nominal electro-optical characteristics at *T*a = 20 °C from the manufacturer datasheet [16].

Part Number	*I*_F_ (mA)	*U*_f_ (V)	Radiant Power (mW)	Wavelength (nm)
OSI3NA5111Y	100	1.6	60	850
OSI5CA5111Y	100	1.6	60	940

**Table 2:** Stability thresholds applied during the steady-state detection sequence.

Phase	LED State	Threshold |*dT*/*dt*|	Confirmation Time	Scope
Pre-Balance	OFF	<0.015 °C/s	15 s	Confirm thermal equilibrium with environment
Measurement	ON	<0.005 °C/s	60 s	Confirm calorimetric balance convergence

**Table 3:** Mean absorbed power (*P*_abs_) as a function of LED drive current for all four configurations.

*I*_set_ (mA)	850 nm/10 mm	850 nm/15 mm	940 nm/10 mm	940 nm/15 mm
	Mean ± Std	Mean ± Std	Mean ± Std	Mean ± Std
30	4.1 ± 0.3	5.0 ± 0.2	3.9 ± 0.1	4.8 ± 0.2
50	6.5 ± 0.1	8.1 ± 0.2	6.3 ± 0.2	7.7 ± 0.3
60	8.3 ± 0.2	10.3 ± 0.2	8.0 ± 0.1	10.1 ± 0.1
70	9.7 ± 0.2	11.7 ± 0.1	9.3 ± 0.3	11.5 ± 0.2
80	11.6 ± 0.3	14.1 ± 0.3	11.1 ± 0.3	13.7 ± 0.1
90	13.0 ± 0.4	15.5 ± 0.7	12.1 ± 0.2	15.0 ± 0.2
100	14.1 ± 0.2	17.2 ± 0.4	13.3 ± 0.3	16.5 ± 0.3

**Table 4:** Linear regression parameters for *P*_abs_ as a function of LED drive current.

Wavelength (nm)	Diameter (mm)	Slope (mW/mA)	Intercept (mW)	*R* ^2^
850	10	0.148	−0.573	0.995
850	15	0.177	−0.457	0.997
940	10	0.138	−0.361	0.996
940	15	0.171	−0.419	0.996

**Table 5:** Linear regression parameters for *P*_abs_ as a function of LED electrical input power.

Wavelength nm	Diameter mm	Slope mW/mW	Intercept mW	*R* ^2^
850	10	0.086	−0.020	0.997
850	15	0.101	0.538	0.9997
940	10	0.084	0.461	0.9995
940	15	0.106	0.476	0.9978

**Table 6:** System capture ratio *η*_cap_ (%) as a function of drive current for all configurations.

Configuration	30 mA	50 mA	60 mA	70 mA	80 mA	90 mA	100 mA
850 nm/10 mm	9.14	8.52	8.35	8.41	8.65	8.78	8.64
850 nm/15 mm	11.23	10.91	10.75	10.54	10.51	10.41	10.49
940 nm/10 mm	9.4	9.02	8.77	8.95	8.72	8.65	8.63
940 nm/15 mm	11.87	11.4	11.34	10.77	10.84	11.03	11.08

**Table 7:** Comparison at *I*_LED_ = 100 mA: experimental absorbed power and electrical input power.

Configuration	*P*_abs_ (mW)	*P*_el_ (mW)	*η*_cap_ %
850 nm/10 mm	14.1	163.0	8.6
850 nm/15 mm	17.2	163.8	10.5
940 nm/10 mm	13.3	154.0	8.6
940 nm/15 mm	16.5	148.7	11.1

**Table 8:** Comparison of thermal-based optical power estimation methods for LED characterization.

Criterion	Strąkowska et al. [6,7], Sensors 2024	Kim et al. [9], Sensors 2021	This Work
Detection method	IR thermographic camera—spatial temperature distribution of LED package	Sheet-type thermocouple pair with photo-absorbent metal film (custom sensor)	Contactless MLX90614 IR thermometer—point temperature of absorbing target
Physical principle	Simultaneous measurement of LED + reference resistor under identical cooling	Differential optical absorption between two thermocouples with different absorptivities	Radiative–convective steady-state energy balance on isolated target
Measured quantity	LED optical power (indirect, via thermal model + electrical power)	Absorbed optical power + junction temperature (simultaneous)	Absorbed optical power (*P*_abs_); capture ratio *η*_cap_ = *P*_abs_/*P*_el_
LED type	Visible LED, standard package	High-power LED (automotive), ~645 mW optical output	Low-power NIR LEDs: 850 nm and 940 nm, 30–100 mA
Absorbed power range	Not specified	High power (~645 mW)	Low power: 1.4–17.2 mW
Spectral range	Visible spectrum	Visible/white LED	Near-infrared: 850–940 nm
Primary sensor	IR thermographic camera (high cost, lab-grade)	Custom-built photothermal sensor (specialized fabrication)	Commercial MLX90614 (~$5–10, off-the-shelf)
Embedded/portable	No—laboratory setup required	Partially—compact sensor, external electronics needed	Yes—fully integrated, microcontroller-based
Absolute calibration	Yes—validated vs. integrating sphere	Yes—validated vs. integrating sphere	No—relative/comparative measurement only
Measurement uncertainty	GUM propagation; temperature uncertainty dominates	±3% max error vs. integrating sphere (at 645 mW)	13–70% (operating-point-dependent); <25% within recommended envelope
Linearity (R^2^)	Not reported	Not reported	0.995–0.999 across all configurations
Repeatability	Validated vs. integrating sphere	Validated vs. integrating sphere	RSD < 5% for most configurations (one exception: 6.5% at 30 mA/10 mm)
Stabilization detection	Not described explicitly	Not described explicitly	Derivative threshold |*d*T/*d*t| < 0.005 °C/s, confirmed for 60 s
Data logging	IR camera software/PC	External PC	On-board microSD, CSV format, 1 Hz

## Data Availability

The data presented in this study are openly available in the GitHub repository at https://github.com/popase/Manuscript_sensors-26-04055, (accessed on 22 June 2026), and within this article and its Supplementary Materials.

## Supplementary Materials

The following supporting information can be downloaded at: https://www.mdpi.com/article/10.3390/s26134055/s1, Section S1: Complete experimental dataset, Table S1: Complete experimental dataset of measured and derived quantities for all LED–target configurations; Section S2: Simplified thermal analysis, Table S2.1–S2.4: Simplified thermal analysis and geometry-dependent heat-transfer coefficients.

## Abbreviations

The following abbreviations are used in this manuscript:

A	Effective area of the absorbing target [mm^2^]
CSV	Comma-separated value (data format)
DIP-switch	Dual in-line package switch (manual current adjustment)
ESP32	Espressif Systems ESP32 microcontroller
FOV	Field of view
I^2^C	Inter-integrated circuit serial communication protocol
IIR	Infinite impulse response (digital filter)
INA219	Current and power monitor sensor (Texas Instruments)
IoT	Internet of things
IR LED	Infrared light-emitting diode
MEMS	Micro-electromechanical system
MLX	MLX90614
MLX90614	Contactless infrared thermometer (Melexis)
NIR	Near-infrared
PLA	Polylactic acid (3D-printing thermoplastic)
RSS	Root-sum-square (uncertainty aggregation method)
SD	Secure Digital (memory card)
SPI	Serial peripheral interface
TFT	Thin-film transistor (display)

## References

[1] Argirusis N., Achilleos A., Alizadeh N., Argirusis C., Sourkouni G. (2025). IR Sensors, Related Materials, and Applications. Sensors.

[2] Rogalski A. (2003). Infrared detectors: Status and trends. Prog. Quantum Electron..

[3] Rogalski A. (2012). History of infrared detectors. Opto-Electron. Rev..

[4] Bao A., Lei C., Mao H., Li R., Guan Y. (2019). Study on a High Performance MEMS Infrared Thermopile Detector. Micromachines.

[5] Pohl T., Meindl P., Johannsen U., Taubert D., Werner L. (2019). Measurement of the absolute spectral responsivity in the mid-infrared based on the cryogenic electrical substitution radiometer and an optimized thermopile detector. J. Sens. Sens. Syst..

[6] Strakowska M., Felczak M., Torzyk B., Shatarah I., Urbas S., Tabaka P. Thermal modeling and measurement of LED optical power using IR thermography. Proceedings of the Quantitative InfraRed Thermography Conference (QIRT).

[7] Strąkowska M., Urbaś S., Felczak M., Torzyk B., Shatarah I.S.M., Kasikowski R., Tabaka P., Więcek B. (2024). Modelling and Thermographic Measurements of LED Optical Power. Sensors.

[8] Modest M.F. (2013). Radiative Heat Transfer.

[9] Kim Y.-Y., Joo J.-Y., Kim J.-M., Lee S.-K. (2021). Compact Measurement of the Optical Power in High-Power LED Using a Light-Absorbent Thermal Sensor. Sensors.

[10] Hegedus J., Hantos G., Lukacs M., Bodnar B., Lipák G., Poppe A. (2022). Thermal characterization issues of LEDs during reliability testing. 2022 28th International Workshop on Thermal Investigations of ICs and Systems (THERMINIC).

[11] Melexis (2019). MLX90614 Infrared Thermometer—Datasheet.

[12] Melexis (2023). MLX90632 Miniature SMD Infrared Thermometer IC—Datasheet.

[13] Lloyd J.R., Moran W.R. (1974). Natural Convection Adjacent to Horizontal Surface of Various Planforms. J. Heat Transf..

[14] Tychanicz-Kwiecień M., Grosicki S., Markowicz M. (2025). Experimental Investigation of Thermal Conductivity of Selected 3D-Printed Materials. Materials.

[15] MatWeb Overview of Materials for Polylactic Acid (PLA) Biopolymer. https://www.matweb.com/search/DataSheet.aspx?MatGUID=ab96a4c0655c4018a8785ac4031b9278.

[16] (2020). 5 mm Round Infrared LED.

[17] Texas Instruments (2015). INA219 Zero-Drift, Bidirectional Current/Power Monitor with I2C Interface. SBOS448G. https://www.ti.com/product/INA219.

[18] Amphenol Advanced Sensors (2024). Thermometrics Thermopile IR Sensor Applications—Application Note.

[19] Hamamatsu Photonics (2024). Thermopile Detectors—Technical Note.

[20] Melexis Distance-to-Spot Size Calculator. https://distance-to-spot.melexis.com/.

[21] Design1st (2024). Thermal Emissivity Values for Engineering Materials.

[22] The Engineering Toolbox (2024). Emissivity Coefficients of Common Engineering Materials. https://www.engineeringtoolbox.com/amp/emissivity-coefficients-d_447.html.

[23] Joint Committee for Guides in Metrology (2008). Evaluation of Measurement Data—Guide to the Expression of Uncertainty in Measurement (GUM).

[24] Piprek J. (2010). Efficiency droop in nitride-based light-emitting diodes. Phys. Status Solidi A.

[25] Wagner M., Beutel B., Naglic P., Fugger O., Foschum F., Kienle A. (2026). Determination of Finger Optical Properties Using an Integrating Sphere. Sensors.

[26] Schwind K.M., Nevas S., Sperfeld P., Pape S. (2024). Characterisation of an LED-based integrating sphere source for detection of changes of AERONET Europe radiometers. J. Phys. Conf. Ser..

[27] Barbosa da Cruz Junior L., Bachmann L. (2020). Manufacture and characterization of a 3D-printed integrating sphere. Instrum. Sci. Technol..

