# Supplementary material for: Indirect Estimation of Absorbed Infrared LED Radiant Power Using Contactless Thermal Sensing
Source: Sensors (Basel). 2026 Jun 26;26(13):4055. doi: 10.3390/s26134055 (PMC13363740; doi:10.3390/s26134055)
Supplement: Supplementary file 1 [file sensors-26-04055-s001.zip › sensors-4377203-supplementary.pdf]

sensors

MDPI

Supplementary Materials

S1. Complete experimental dataset

For all four configurations Table S1 presents all intermediate quantities.

Table S1. Steady-state experimental results for all LED–target configurations and drive currents. Values are reported as mean ± standard deviation across five independent repetitions.

λ (nm)	d (mm)	I_{set} (mA)	I_{LED} (mA)	U_{LED} (V)	T_{amb} (°C)	ΔT (°C)	P_{abs} (mW)	P_{conv} (mW)	P_{rad} (mW)	η_{cap} (%)
		30	29.33 ± 0.01	1.54 ± 0.01	24.85 ± 0.34	1.6 ± 0.1	4.1 ± 0.3	2.7 ± 0.2	1.5±0.1	9.1 ± 0.6
		50	47.00 ± 0.04	1.62 ± 0.01	24.52 ± 0.53	2.49 ± 0.04	6.5 ± 0.1	4.2 ± 0.1	2.3±0.1	8.5 ± 0.2
		60	59.66 ± 0.03	1.66±0.01	24.65±0.16	3.17±0.06	8.3±0.2	5.3±0.1	2.9±0.1	8.3±0.1
850	10	70	69.09±0.01	1.67±0.01	22.91±0.14	3.73±0.09	9.7±0.2	6.3±0.2	3.4±0.1	8.4±0.2
		80	82.59±0.01	1.62±0.01	23.20±0.10	4.4±0.1	11.6±0.3	7.5±0.2	4.1±0.1	8.7±0.2
		90	91.34±0.02	1.62±0.01	23.08±0.43	5.0±0.2	13.0±0.4	8.4±0.3	4.6±0.1	8.8±0.3
		100	100.31±0.01	1.625±0.01	23.49±0.15	5.4±0.1	14.1±0.2	9.1±0.2	5.0±0.1	8.6±0.1
		30	29.34±0.01	1.526±0.01	24.48±0.07	1.07±0.05	5.0±0.2	2.8±0.1	2.2±0.1	11.2±0.5
		50	47.09±0.01	1.5744±0.01	24.47±0.04	1.72±0.04	8.1±0.2	4.6±0.1	3.5±0.1	10.9±0.2
		60	59.79±0.01	1.5961±0.01	24.41±0.09	2.18±0.04	10.3±0.2	5.8±0.1	4.5±0.1	10.8±0.2
850	15	70	69.28±0.01	1.608±0.01	24.46±0.02	2.49±0.02	11.7±0.1	6.6±0.1	5.1±0.1	10.5±0.1
		80	82.61±0.01	1.624±0.01	24.05±0.21	3.00±0.06	14.1±0.3	7.9±0.2	6.2±0.1	10.5±0.2
		90	91.29±0.03	1.630±0.01	22.53±0.21	3.3±0.2	15.5±0.7	8.8±0.4	6.7±0.3	10.4±0.5
		100	100.25±0.04	1.63±0.01	22.67±0.32	3.67±0.07	17.2±0.4	9.7±0.2	7.5±0.2	10.5±0.2
		30	29.630±0.006	1.3840±0.0001	23.70±0.09	1.49±0.04	3.9±0.1	2.5±0.1	1.4±0.1	9.4±0.2
		50	47.26±0.02	1.475±0.01	22.51±0.35	2.43±0.06	6.3±0.2	4.1±0.1	2.2±0.1	9.0±0.3
		60	59.90±0.02	1.53±0.02	23.04±0.31	3.10±0.04	8.0±0.1	5.2±0.1	2.8±0.1	8.8±0.2
940	10	70	69.53±0.01	1.49±0.01	24.28±0.45	3.6±0.1	9.3±0.3	6.0±0.2	3.3±0.1	8.9±0.3
		80	82.84±0.03	1.53±0.01	24.52±0.256	4.2±0.1	11.1±0.3	7.1±0.2	3.9±0.1	8.7±0.2
		90	91.64±0.02	1.53±0.01	24.84±0.13	4.63±0.09	12.1±0.2	7.8±0.1	4.3±0.1	8.7±0.2
		100	100.50±0.02	1.53±0.01	22.98±0.29	5.1±0.1	13.3±0.3	8.6±0.2	4.7±0.1	8.6±0.2
		30	29.70±0.01	1.36±0.01	23.67±0.33	1.03±0.04	4.8±0.2	2.7±0.1	2.1±0.1	11.9±0.5
		50	47.41±0.03	1.43±0.02	24.62±0.19	1.65±0.06	7.7±0.3	4.4±0.2	3.4±0.1	11.4±0.5
		60	60.06±0.05	1.48±0.02	24.55±0.23	2.14±0.03	10.1±0.1	5.7±0.1	4.4±0.1	11.3±0.2
940	15	70	69.40±0.01	1.55±0.01	23.80±0.20	2.46±0.04	11.5±0.2	6.5±0.1	5.0±0.1	10.8±0.2
		80	82.85±0.03	1.52±0.01	24.31±0.07	2.90±0.03	13.7±0.1	7.7±0.1	6.0±0.1	10.8±0.1
		90	91.67±0.01	1.49±0.01	24.15±0.08	3.19±0.04	15.0±0.2	8.5±0.1	6.6±0.1	11.0±0.1
		100	100.64±0.03	1.48±0.01	23.53±0.45	3.51±0.06	16.5±0.3	9.3±0.2	7.2±0.2	11.1±0.2

S2. Simplified thermal analysis

This supplementary section provides the simplified thermal analysis used to estimate the geometry-dependent effective heat-transfer coefficients adopted in the main manuscript. The calculations are based on characteristic-length Rayleigh–Nusselt correlations for small horizontal discs operating in the low-Rayleigh-number regime.

Rayleigh numbers computed from mean experimental ΔT and T_{amb} per operating point. $L_c = D/4$ (Lloyd–Moran); $Nu = \max(1, 0.54 \cdot Ra0.25)$; $h = Nu \cdot k_{air}/L_c$, $k_{air} = 0.02625$ W/m·K. All $Ra \ll 2.2\times10^4$ (conduction-dominated regime).

- h_{10} for 10 mm target → 10.5 W/m²K ($Nu \approx 1.000$ under low-Rayleigh conditions);
- h_{15} for 15 mm target → 7.4 W/m²K (adopted representative value; operating-point range 7.00–7.82 W/m²K, arithmetic mean 7.28 W/m²K).

Tables S2.1 and S2.2 present centralized data for calculated h_{10} and h_{15}.

Table S2.1 — 850 nm / 10 mm; (L_c = 2.5 mm, A = 160.221 mm², h_{10} = 10.5 W/m²K).

I [mA]	*Ra*	*Nu*	h_{calc} [W/m²K]	h_{10} [W/m²K]	Diff [%]	ΔP_{abs} [%]
30	2.33	1.0000	10.500	10.5	0.00%	0.0%
50	3.67	1.0000	10.500	10.5	0.00%	0.0%
60	4.66	1.0000	10.500	10.5	0.00%	0.0%
70	5.51	1.0000	10.500	10.5	0.00%	0.0%
80	6.55	1.0000	10.500	10.5	0.00%	0.0%
90	7.36	1.0000	10.500	10.5	0.00%	0.0%
100	7.94	1.0000	10.500	10.5	0.00%	0.0%

Table S2.2 — 850 nm / 15 mm (L_c = 3.75 mm, A = 358.142 mm², h_{15} = 7.4 W/m²K)

I [mA]	*Ra*	*Nu*	h_{calc} [W/m²K]	h_{15} [W/m²K]	Diff [%]	ΔP_{abs} [%]
30	5.33	1.0000	7.000	7.4	-5.41%	-3.0%
50	8.56	1.0000	7.000	7.4	-5.41%	-3.0%
60	10.85	1.0000	7.000	7.4	-5.41%	-3.0%
70	12.40	1.0132	7.093	7.4	-4.15%	-2.3%
80	14.91	1.0611	7.428	7.4	+0.38%	+0.2%
90	16.55	1.0891	7.624	7.4	+3.03%	+1.7%
100	18.31	1.1171	7.819	7.4	+5.67%	+3.1%

The observed deviations remain small relative to the overall experimental uncertainty of the proposed comparative calorimetric methodology.

Table S2.3 — 940 nm / 10 mm (L_c = 2.5 mm, A = 160.221 mm², h_{10} = 10.5 W/m²K)

I [mA]	R_a	*Nu*	h_{calc} [W/m²K]	h_{10} [W/m²K]	Diff [%]	ΔP_{abs} [%]
30	2.20	1.0000	10.500	10.5	0.00%	0.0%
50	3.61	1.0000	10.500	10.5	0.00%	0.0%
60	4.58	1.0000	10.500	10.5	0.00%	0.0%
70	5.24	1.0000	10.500	10.5	0.00%	0.0%
80	6.22	1.0000	10.500	10.5	0.00%	0.0%
90	6.78	1.0000	10.500	10.5	0.00%	0.0%
100	7.53	1.0000	10.500	10.5	0.00%	0.0%

Table S2.4 – 940 nm / 15 mm (L_c = 3.75 mm, A = 358.142 mm², h_{15} = 7.4 W/m²K)

I [mA]	*Ra*	*Nu*	h_{calc} [W/m²K]	h_{15} [W/m²K]	Diff [%]	ΔP_{abs} [%]
30	5.13	1.0000	7.000	7.4	-5.41%	-3.0%
50	8.19	1.0000	7.000	7.4	-5.41%	-3.0%
60	10.64	1.0000	7.000	7.4	-5.41%	-3.0%
70	12.25	1.0102	7.072	7.4	-4.44%	-2.4%
80	14.41	1.0520	7.364	7.4	-0.48%	-0.3%
90	15.87	1.0778	7.545	7.4	+1.95%	+1.1%
100	17.46	1.1038	7.727	7.4	+4.41%	+2.4%

Cells shaded red: |Diff| > 5% (15 mm disc, high currents). ΔP_{abs} estimated as Diff × (P_{conv}/P_{abs}) ≈ Diff × 0.56, since convection contributes ~56% of P_{abs}. Maximum |ΔP_{abs}| < 3.2% - within combined uncertainty budget. Rayleigh numbers computed for the 940 nm dataset yield h_{calc} values differing by less than 1.2% from those reported in Tables S1.1–S1.2, with differences below 0.5% for drive currents up to 70 mA, confirming that the adopted coefficients are geometry-dependent rather than wavelength-dependent.
